# Supplementary material for: Assessment of Nutritional Status and Nutrition Impact Symptoms in Patients Undergoing Resection for Upper Gastrointestinal Cancer: Results from the Multi-Centre NOURISH Point Prevalence Study
Source: Nutrients. 2021 Sep 24;13(10):3349. doi: 10.3390/nu13103349 (PMC8539371; doi:10.3390/nu13103349)
Supplement: Supplementary file 1 [file nutrients-13-03349-s001.zip › nutrients-1371093-supplementary.pdf]

Supplementary Table S1. Unintentional Weight Loss by Surgery Type[a]

Variable	Overall		Oesophagectomy		Gastrectomy		Pancreatectomy		P value
**% LOW in > 6 months** [b]	**8.3**	**4.3, 12.5**	**8.4**	4.8, 15.1	8.7	5.5, 14.1	8.3	4.3, 11.9	0.531
**% LOW in 6 months** [b]	7.4	3.8, 11.6	8.2	3.2, 12.7	5.2	3.6, 10.0	7.8	4.2, 11.5	0.647
**% LOW in 3 months** [b]	4.8	1.8, 8.2	5.0	1.4, 6.7	3.1	0.0, 7.2	5.1	3.3, 9.6	0.084
**% LOW in 1 month** [b]	1.3	0, 3.2	0.5	0.0, 2.5	0.0	0.0, 1.9	2.2	0.0, 4.8	**0.021**
**% LOW in 2 weeks** [b]	0	0, 1.7	0.0	0.0, 1.0	0.0	0.0, 1.2	0.0	0.0, 2.5	0.268
**LOW ≥5% in 2 weeks (n, %)**									0.434
No	175	95.6	60	96.8	45	97.8	70	93.3	
Yes	8	4.4	2	3.2	1	2.2	5	6.7	
**LOW ≥5% in 1 month (n, %)**									0.299
No	165	90.7	60	93.8	40	93.0	65	86.7	
Yes	17	9.3	4	6.3	3	7.0	10	13.3	
**LOW ≥5% in 3 months (n, %)**									0.665
No	128	69.6	43	68.3	33	75.0	52	67.5	
Yes	56	30.4	20	31.7	11	25.0	25	32.5	
**LOW ≥5% in 6 months (n, %)**									0.588
No	109	58.0	37	56.9	29	64.4	43	55.1	
Yes	79	42.0	28	43.1	16	35.6	35	44.9	
**LOW ≥5% in > 6 months (n, %)**									0.943
No	95	51.1	32	51.6	23	48.9	40	51.9	
Yes	91	48.9	30	48.4	24	51.1	37	48.1	
**LOW ≥10% in 2 weeks (n, %)**									-
No	183	100	62	100	46	100	75	100	
Yes	0	0	0	0	0	0	0	0	
**LOW ≥10% in 1 month (n, %)**									0.272
No	176	97.8	64	100	41	9.6	71	95.9	
Yes	4	2.2	0	0	1	2.4	3	4.1	
**LOW ≥10% in 3 months (n, %)**									0.270
No	163	90.6	57	91.9	41	95.3	65	86.7	
Yes	17	9.4	5	8.1	2	4.7	10	13.1	
**LOW ≥10% in 6 months (n, %)**									0.401
No	140	77.8	47	74.6	35	85.4	58	76.3	
Yes	40	22.2	16	25.4	6	14.6	18	23.7	
**LOW ≥10% in > 6 months (n, %)**									0.898
No	126	72.8	43	71.7	31	75.6	52	72.2	
Yes	47	27.2	17	28.3	10	24.4	20	27.8	

[a] Data reported for those with weight data available for each timeframe, missing data excluded.

[b] Presented as median (IQR). LOW=Loss of Weight
